# 3D and Inkjet Printing by Colored Mie-Resonant Silicon Nanoparticles Produced by Laser Ablation in Liquid

**DOI:** 10.3390/nano13060965

**Published:** 2023-03-07

**Authors:** Lev Logunov, Aleksandr Ulesov, Vladislava Khramenkova, Xiuzhen Liu, Aleksandr A. Kuchmizhak, Alexander Vinogradov, Sergey Makarov

**Affiliations:** 1 School of Physics and Engineering, ITMO University, Saint Petersburg 191002, Russia; 2 SCAMT, ITMO University, Saint Petersburg 191002, Russia; 3 Qingdao Innovation and Development Center, Harbin Engineering University, Qingdao 266000, China; 4 Institute for Automation and Control Processes, Far Eastern Branch of the Russian Academy of Sciences, Vladivostok 690041, Russia; 5 Far Eastern Federal University, Vladivostok 690922, Russia; 6 Institute of Chemistry, Saint Petersburg State University, 26 Universitetskii pr, Saint Petersburg 198504, Russia

**Keywords:** laser ablation, nanoparticles, silicon, printings, Mie resonances, coloring

## Abstract

Optically resonant silicon nanoparticles have emerged as a prospective platform for the structural coloration of surfaces because of their strong and spectrally selective light scattering. In this work, we developed colorful inks based on polymer mixed with monodisperse Mie-resonant silicon nanoparticles for 3D and inkjet printing. We applied a laser ablation method in a flow cell for the mass production of silicon nanoparticles in water and separated the resulting nanoparticles with different sizes by density-gradient centrifugation. Mixing the colorful nanoparticles with the polymer allows for the printing of 3D objects with various shapes and colors, which are rigid against environmental conditions.

## 1. Introduction

Nanoparticles can provide structural colors that emerge from resonant interactions with light [1]. Plasmonic nanoparticles were one of the main platforms because of their fabrication simplicity for resonant metal nanoparticles [2]. Aside from the extremely high cost of gold, plasmon resonances are restrained to a range of colors, from the green to red end of the spectrum. Aluminum, while cost-efficient and able to support resonances throughout the visible spectrum, suffers from broad resonances that hinder color pureness and saturation [3]. Silver may provide the most optimal optical properties for coloration but suffers from limited stability in ambient conditions.

In turn, all-dielectric nanophotonics [4,5,6] offer unique opportunities for the structural coloring of various surfaces based on the strong scattering of Mie-resonant nanoparticles made of high-index materials, such as silicon, which is extremely stable in ambient conditions. The first silicon nanoparticle realization of coloring was demonstrated on a microscale based on single-particle laser printing [7] or lithography-based approaches [8]. However, developing printable inks based on nanoparticles mixed with liquids to develop up-scalable printing technologies for Mie-resonant coloring is still challenging [9,10].

In this work, we developed inks based on Mie-resonant silicon nanoparticles for 3D and inkjet printing applications. To achieve this goal, we applied a high-throughput laser ablation method to produce hundreds of milliliters of silicon nanoparticles in water, and then we separated the nanoparticles of different sizes by a density-gradient centrifugation approach. The nanoparticles’ morphology and optical characterization, which was supported by modeling, reveal their excellent quality for further application in coloration and inkjet printing. Proof-of-concept large-scale printing by inks with the obtained silicon nanoparticles is demonstrated in this paper for the first time.

## 2. Results and Discussion

### 2.1. Large-Scale Laser Ablation Synthesis

For the synthesis of silicon nanoparticles, we used the laser ablation in liquid (LAL) method. There are several advantages to using the LAL method compared with the chemical or physical methods of nanoparticle synthesis: (1) LAL is a continuous synthesis method that allows one to scale the production volume in an understandable and accessible way; (2) the LAL method results in pure nanoparticles without the presence of contaminants; thus, the nanoparticles are ready to use immediately after synthesis; (3) the method is environmentally friendly due to the absence of chemical reagents, which are usually required in the synthesis of nanoparticles by chemical technologies. Moreover, the LAL method has a low carbon footprint, especially when using modern laser sources. (4) The laser ablation reaction is carried out in a sealed environment, which prevents human contact with nanoparticles. For example, when using mechanical grinding, airborne particles can be inhaled into the human body, creating a threat associated with the biotoxicity of the particles. (5) LAL is an “easy to learn and use” technique that does not require a long period of training or a long period of knowledge accumulation; (6) having a reactor for carrying out the LAL reaction, you can both change the material for ablation and choose the solvent in which you want to obtain nanoparticles; (7) the LAL method gives the ability to control the shape of synthesized nanoparticles.

**Figure 1:**
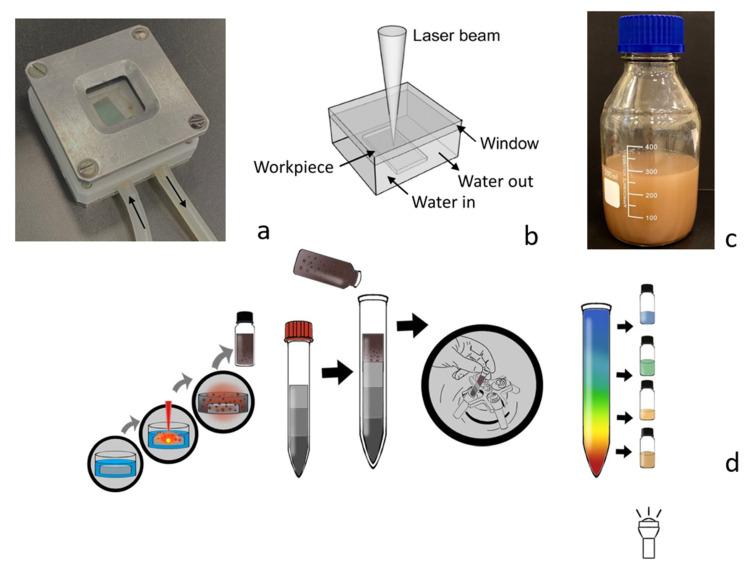
Method and principle of separation. (**a**) Photography of a flow cell with an optical window for laser focusing on a silicon wafer. The black arrows indicate water flow directions. (**b**) Scheme of the flow cell. (**c**) Bottle with the solution of silicon nanoparticles of a volume of around 300 mL, generated by approximately 3 h. (**d**) Schematic illustration of the density-gradient centrifugation method: starting from laser generation of nanoparticles, then mixing with sucrose, then centrifugation, resulting in separation of the nanoparticles, and, finally, redistribution of the different nanoparticles by sucking them from different levels of the cuvette.

The process of the synthesis and nanoparticle separation is presented in Figure 1. We used femtosecond laser irradiation with a wavelength of 1040 nm, frequency of 1 MHz, and pulse energy of 10 μJ. Irradiation was focused on the spot (20 μm) by an objective 10x. For the mass production of nanoparticles, we created a flow cell, which was made from fluoroplastic. For the window, we used quarts of glass with a 4-mm thickness, the volume of the cell was 1.5 mL, and the volume of the whole system was 100 mL with a flow speed of 3 mL/s. The photo and scheme of the cell are presented in Figure 1a,b. The cell was moved by a motorization setup, and the speed of scanning was 3 mm/s.

The nanoparticle generation was stopped when the solution became brown and achieved enough volume for further experiments (Figure 1c). The concentration of the silicon nanoparticles in water was measured via dry residue measurement and by measuring the optical absorbance of the solution in the visible range and calculating using the Mie theory. The reaction yield was about 10 mg/100 mL per 1 h. The size distribution of the nanoparticles in the stock solution is shown in Supplementary Materials (Figure S1).

### 2.2. Size Separation Technique

In order to separate a pristine colloidal dispersion into solutions of size-purified Si NP, we employed a sucrose density-gradient centrifugation process. The sucrose density-gradient solutions were prepared by carefully adding 2 mL of the sucrose solutions at five different concentrations (60, 50, 40, 30 and 20 wt%) to a 15-mL centrifugal tube in order. The colloidal dispersion of Si NP (1 mL) was added to the top of the tube, and the tube was subjected to centrifugation at 4500 rpm for 25 min to form layers of size-separated Si NSs. The color changed from blue to orange from top to bottom. By collecting small amounts of the solutions from the top, we obtained solutions of different colors. The layers were retrieved from the top and transferred to different vials. The solutions of size-separated Si NSs were washed with water and methanol several times to remove sucrose. For the mass production of size-purified SiNP, we paralleled the process to 12 centrifugal tubes (Figure S2.). As a result, we obtained 12 mL of five colored solutions (see Figure 1d).

**Figure 2:**
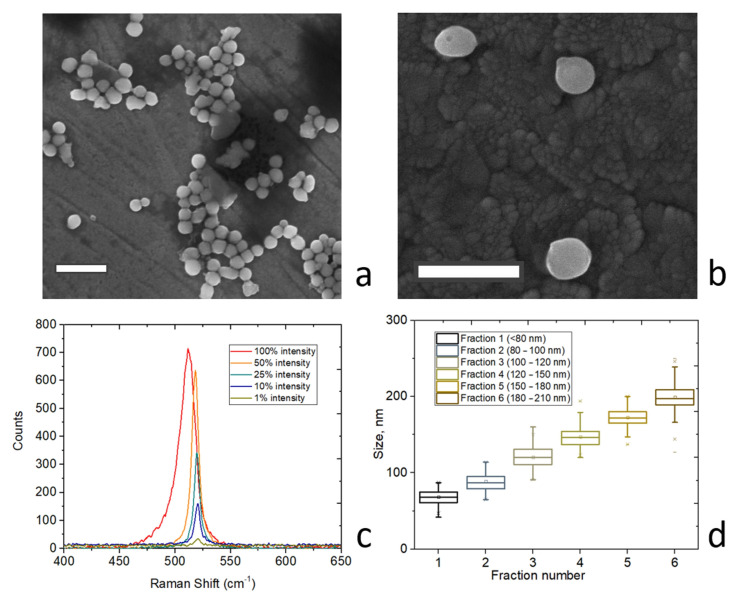
Nanoparticles characterization. (**a**) SEM image of silicon nanoparticles on ITO with diameters of 120–150 nm. Scale bar is 500 nm. (**b**) SEM image of silicon nanoparticles on ITO with diameters of 180–210 nm. Scale bar is 500 nm. (**c**) Raman spectrum silicon nanoparticle at different incident powers from 40 μW to 4 mW. (**d**) Size distributions for six different fractions of silicon nanoparticles.

Figure 2a shows a scanning electron microscopy (SEM) image of the produced nanoparticles after the centrifugation procedure. One can see that these nanoparticles possess almost the same size, around *d* = 120–150 nm. Higher magnification of the larger particles (*d* = 180–210 nm) reveals their smooth surface and regular shape with some ellipticity, which does not significantly affect the low-order Mie modes [11]. Additionally, according to previous studies, the nanoparticle surface is covered by a few-nm silicon oxide layer due to the natural oxidation process [11], while the internal structure represents nanograins with a developed network of interfaces [12].

In Figure 2c, the Raman spectra of a silicon nanoparticle (d = 160 nm), measured upon CW laser irradiation (wavelength 633 nm, maximum power 4 mW, focused by an objective NA = 0.65) on glass, confirms a good crystallinity of the nanoparticles. The Raman spectra measurements were carried out by utilizing a spectrometer (LabRam HR, HORIBA, Japan) with a 600 g/mm diffraction grating. The observed Raman peak of around 520 cm^−1^ corresponds to a typical c-Si material, while the intensity is strong enough for observation, even by a signal acquiring less than 1 s because of the laser-matter interaction enhancement with Mie resonances [13]. Remarkably, the peak of the Raman spectrum exhibits a gradual spectral shift with elevated laser intensity. This effect is associated with the optical heating of a single silicon nanoparticle on the glass surface, which was observed and described in other studies [13,14].

Additionally, based on the careful and multiple SEM measurements, we revealed that the statistical size distribution is less than 10% of the average (Figure 2d). It means that we can control not only the diameters of the nanoparticle but also their resonant optical properties, which can be employed for coloration.

### 2.3. Optical Characterization and Modeling

**Figure 3:**
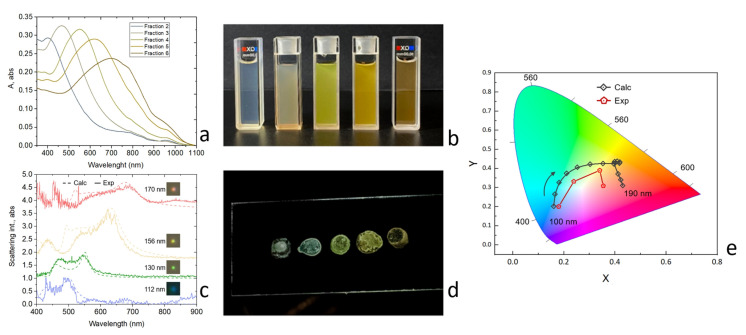
Experimental color effect. (**a**) Absorption spectra of solutions of size-separated Si NP (**b**) Photograph of solutions of size-separated Si NP (**c**) Scattering and dark-field spectra of single Si NP with different diameters (110–170 nm in diameter) placed on a glass substrate. (**d**) Photograph of dried drops of Si NP on glass (**e**) CIE1931 chromaticity diagram with experimental color space values obtained from scattering spectra of single Si NSs of different diameters (100–190 nm) and calculated scattering spectra.

Figure 3a shows the extinction spectra of the colloidal solutions with silicon nanoparticles in water measured by a spectrophotometer (Shimadzu UV-3600 Plus, Shimadzu Ltd., Japan). All these spectra exhibit a similar feature related to the single peak, which is dependent on the average size. Namely, the particles with d = 100–120 nm correspond to a peak of around 465 nm, while the largest particles with d = 170–200 nm provide a peak of around 700 nm. This peak provides specific colors (from blue to red) of the solutions under white light-emitting diode (LED) illumination, as shown in Figure 3b. These colors are a clear indication of the existence of the Mie resonances in the nanoparticles [9,10].

In order to show the Mie resonances clearly in our nanoparticles, we carried out confocal single-particle dark-field micro-spectroscopy experiments. For the measurements, we deposited a drop of the size-purified solution on a cover glass and air-dried them. Figure 3c shows the backward scattering spectra and corresponding scattering images of the different sizes of single Si nanoparticles. These dark-field spectra were measured in a dark-field scheme, where a white light source (HL-2000 halogen lamp, Ocean Optics Ltd., USA) was focused using an infinity-corrected objective with a numerical aperture of NA = 0.26 for the illumination of the sample under a 67 ° angle to the surface normal. The second objective accomplished the scattering signal collection (NA = 0.42) placed perpendicularly to the sample surface. The scattered signal was analyzed by a confocal system with a spectrometer (LabRam HR, HORIBA, Japan) and a cooled CCD camera equipped with a 150 g/mm diffraction grating. The location of the nanoparticles on the glass substrate was controlled by an additional CCD camera. The Mie resonances in individual Si nanoparticles manifested in the scattering spectra shown in Figure 3c. Typically, each nanoparticle exhibits two strong peaks in scattering—so-called electric and magnetic resonances [5,11,15]—which are shifted to the longer wavelength’s spectral range with the increase in diameter. These maxima make the nanoparticles colorful, and the color is strongly dependent on the particle size, similar to the case of colloidal solutions. Dried droplets of these nanoparticles on a glass substrate preserve the color difference observed for individual particles (Figure 3d). Figure 3e shows the comparison of the scattering spectra on a color space CIE 1931 with those calculated by the Mie theory, where one can find a reasonable agreement between the experiment and calculations. The colors of the silicon nanoparticles with diameters from 95 to 200 nm cover a wide area of the sRGB area on the chromaticity diagram.

**Figure 4:**
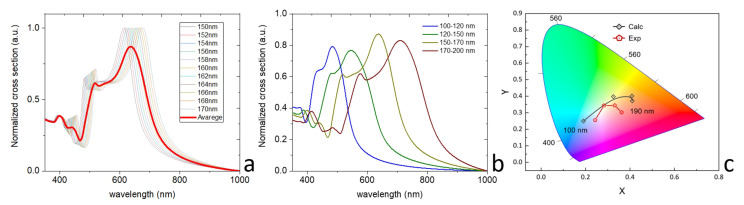
Modeling. (**a**) Calculated extinction spectra for silicon nanoparticles of different sizes (step 2nm) in water (*n* = 1.33); (**b**) Averaged spectra for colored fractions of aqueous solutions Si NP; (**c**) CIE1931 chromaticity diagram with experimental color space values obtained from extinction spectra of the solutions with different NP diameters (100–190 nm) and calculated extinction spectra.

Theoretical modeling of the scattering spectra was carried out by means of the Mie theory [16], implying an analytical solution for a single spherical nanoparticle irradiated by a plane wave. The silicon refractive index was taken from the literature [17], while the host media was varied depending on the conditions. In Figure 4a, we show the calculated extinction spectra for the silicon nanoparticles of different sizes in water (*n* = 1.33), which qualitatively agrees with our dark-field measurements in Figure 3c. Moreover, we calculated the averaged extinction spectrum for the range of nanoparticles from *d* = 150 nm to 170 nm, which can also be performed for the different ranges of particle diameters from Figure 3a. The theoretical calculations confirm that the increase in particle diameter results in a red shift of the resonances and, thus, a strong shifting of coordinates on the color space. The theoretical and experimental extinction spectra of the solutions are compared directly on a single color space CIE 1931 in Figure 4c. One can clearly see that changing the Si NP average size from 120 to 190 nm can potentially achieve broad color tunability. It means that Si nanoparticles in a liquid environment can be used for the development of inks for printing.

### 2.4. Preparation of Inks and 3D Printing

The inks for the 3D printing were prepared from an aqueous solution of nanoparticles and a solution of PVA (mass fraction 20%), with an 88% degree of hydrolysis (grade 05–88), produced by NevaReaktiv, by mixing in a ratio of 1:1, and evaporation (at 70 °C) at constant stirring (100 rpm) until the solution had the necessary viscosity for printing. After that, the solution was heated to 90 °C, poured into a mold, and left for 10 min under vacuum (desiccator with a vacuum pump) to remove air bubbles from the polymer mass. The finished polymer mass was reheated without stirring to 90 °C and transferred to a 3 mL CELLINK cartridge for 3D printing.

The ink used for jet printing was prepared from an aqueous solution of nanoparticles with the addition of functional additives to adjust the rheological properties of the solution. In the first step, the solution with nanoparticles was passed through a Biofil filter (0.45 µm), after which the filtered solution was modified with the following additives: Polyvinyl alcohol (0.1–3% wt.) and Isopropyl alcohol (1–10% vol.).

Three-dimensional printing was carried out with the synthesized polymer ink based on a solution comprising particles of two colors (green, d = 120–150 nm; blue, d = 100–120 nm) on a BIO X printer from CELLINK. The following printing parameters were used: pressure 10–30 kPa, print speed 1–5 mm/sec, cartridge temperature 50–55 °C. Printing was carried out on a glass substrate, and after printing, the structure was cooled to room temperature and stored in a sealed box.

Three-dimensional printing was carried out with a prepared polymeric ink based on a solution comprising particles of two colors (green and blue) on a BIO X printer from CELLINK with the following printing parameters: pressure 10–30 kPa, print speed 1–5 mm/s, cartridge temperature 50–55 °C. Printing was carried out on a glass substrate, and after printing was completed, the structure was cooled to room temperature and stored in a sealed box.

**Figure 5:**
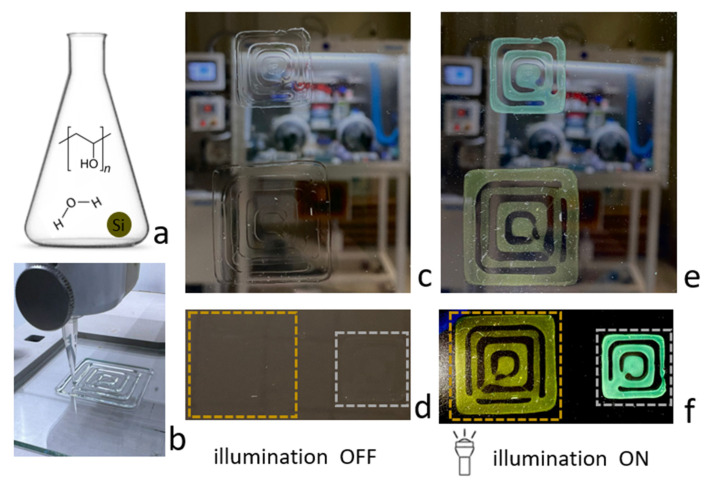
3D printing. (**a**) Ink compositions for 3D printing; (**b**) printing process on glass by green fraction Si NP (d = 120–150nm). (**c**) Printed structures on glass without background, illumination off. (**d**) Printed structures on glass with black background, illumination off. (**e**) Illumination on. (**f**) Illumination on.

A schematic representation of the ink and the 3D printing process itself is shown in Figure 5a,b. Figure 5c,d show the 3D printed structures on glass with ink, with blue (d = 100–120 nm) and green (d = 120–150 nm) fractions under room lighting. As can be seen from the photographs, the printed structures are transparent and practically invisible when using a black background. Whereas with additional white illumination (Figure 5e,f), the structures that use a black background have a more pronounced color.

**Figure 6:**
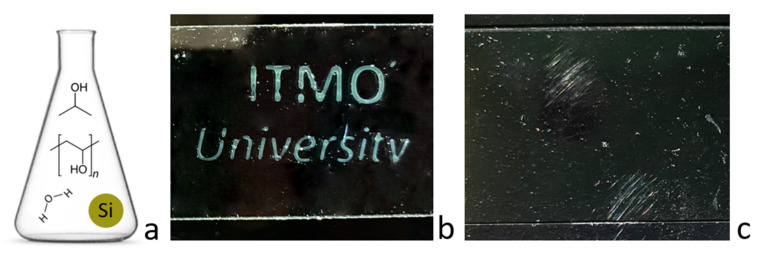
Inkjet printing. (**a**) Ink composition for jet printing; (**b**) printed structures on glass by blue inks (d = 100–120nm), illumination on. (**c**) Illumination off.

The inkjet printing was carried out using a printer (Canon TS 300, CANON INC, Vietnam) employing a Canon PG-445 cartridge, which was pre-washed in distilled water using ultrasound and filled with a prepared ink solution. Printing was carried out in the standard mode on a prepared glass substrate from a glass slide with a functionalized surface (a thin layer of PVA was applied). In order to obtain the thickness of the image layer with a pronounced coloration, printing was carried out in four layers (Figure 6).

## 3. Conclusions and Outlook

In this paper, we developed a relatively low-cost and high-throughput approach to colorful ink fabrication based on the Mie-resonant silicon nanoparticles generated by laser ablation with further density-gradient centrifugation. The created inks were applied for proof-of-concept 3D and inkjet printing. Our optical characterization experiments and supporting models have shown that the obtained colors originate from Mie resonances in each silicon nanoparticle, and thus, they are very robust with time in any environment (air, water, polymer).

The proposed technology, which is based on laser ablation in liquid (LAL), paves the way for the inkjet printing of large-scale polymer patterns with stable colors on arbitrary surfaces or even optoelectronic devices [18,19], and we believe that this approach is promising for industrial applications when all parameters of LAL are optimized for a particular task. For example, the productivity of the LAL method is one of the most important factors for a wide practical application. The productivity of LAL is determined by many factors [20], including power density, ablation threshold, emission wavelength, laser pulse duration, scanning speed, repetition rate, position and shape of a focal point, and other physical and chemical factors. As a rule, productivity increases with the increasing intensity of laser radiation, but gradually, productivity reaches saturation as the synthesized colloidal solution begins to absorb laser radiation energy. This leads to a decrease in productivity and additional fragmentation of the already obtained nanoparticles [21,22]. Additionally, if the threshold value of laser intensity is overcome, one will induce water breakdown, and the performance drops sharply due to the blocking and scattering effects of the bubbles. The composition of the liquid also significantly affects the performance of the LAL method. The most common solvents are water and organic solvents. Additionally, the productivity of synthesis in water is usually much higher than in organic solvents [23], as evidenced by the decrease in the ablation rate with increasing ethanol concentrations in water–ethanol mixtures [24].

As a result, since relatively cheap turnkey femtosecond laser systems are already available on the market, LAL allows for providing a synthesis of nanomaterials without specialized knowledge about the synthesis process, which opens up the possibility of synthesizing nanoparticles at the point of consumption, which is important for materials that cannot be stored for a long duration. Therefore, we envision further development of integrating the LAL method with 3D and inkjet printing technologies, where the creation of new inks with desirable properties is highly important.

## Data Availability

The data presented in this study are available on request from the corresponding author.

## Supplementary Materials

The following supporting information can be downloaded at: https://www.mdpi.com/article/10.3390/nano13060965/s1. Figure S1: The size distribution of Si nanoparticles measured by SEM; Figure S2: The tubes before centrifugation for mass separation of Si nanoparticles.
