# Supplementary material for: 3D and Inkjet Printing by Colored Mie-Resonant Silicon Nanoparticles Produced by Laser Ablation in Liquid
Source: Nanomaterials (Basel). 2023 Mar 7;13(6):965. doi: 10.3390/nano13060965 (PMC10058803; doi:10.3390/nano13060965)
Supplement: Supplementary file 1 [file nanomaterials-13-00965-s001.zip › nanomaterials-2227403-supplementary.pdf]

nanomaterials

Supplementary Materials

3D and Inkjet Printing by Colored Mie-Resonant Silicon Nanoparticles Produced by Laser Ablation in Liquid

Lev Logunov [1,*], Aleksandr Ulesov [2], Vladislava Khramenkova [2], Xiuzhen Liu [3], Aleksandr A. Kuchmizhak [4,5,6], Alexander Vinogradov [2] and Sergey Makarov [1,3,*]

1 School of Physics and Engineering, ITMO University, Saint Petersburg 191002, Russia
2 SCAMT, ITMO University, Saint Petersburg, Russia
3 Qingdao Innovation and Development Center, Harbin Engineering University, Qingdao 266000, China
4 Institute for Automation and Control Processes, Far Eastern Branch of the Russian Academy of Sciences, Vladivostok 690041, Russia
5 Far Eastern Federal University, Vladivostok 690922, Russia
6 Institute of Chemistry, Saint Petersburg State University, 26 Universitetskii pr, Saint Petersburg 198504, Russia
* Correspondence: lev.logunov@metalab.ifmo.ru (L.L.); s.makarov@metalab.ifmo.ru (S.M.)

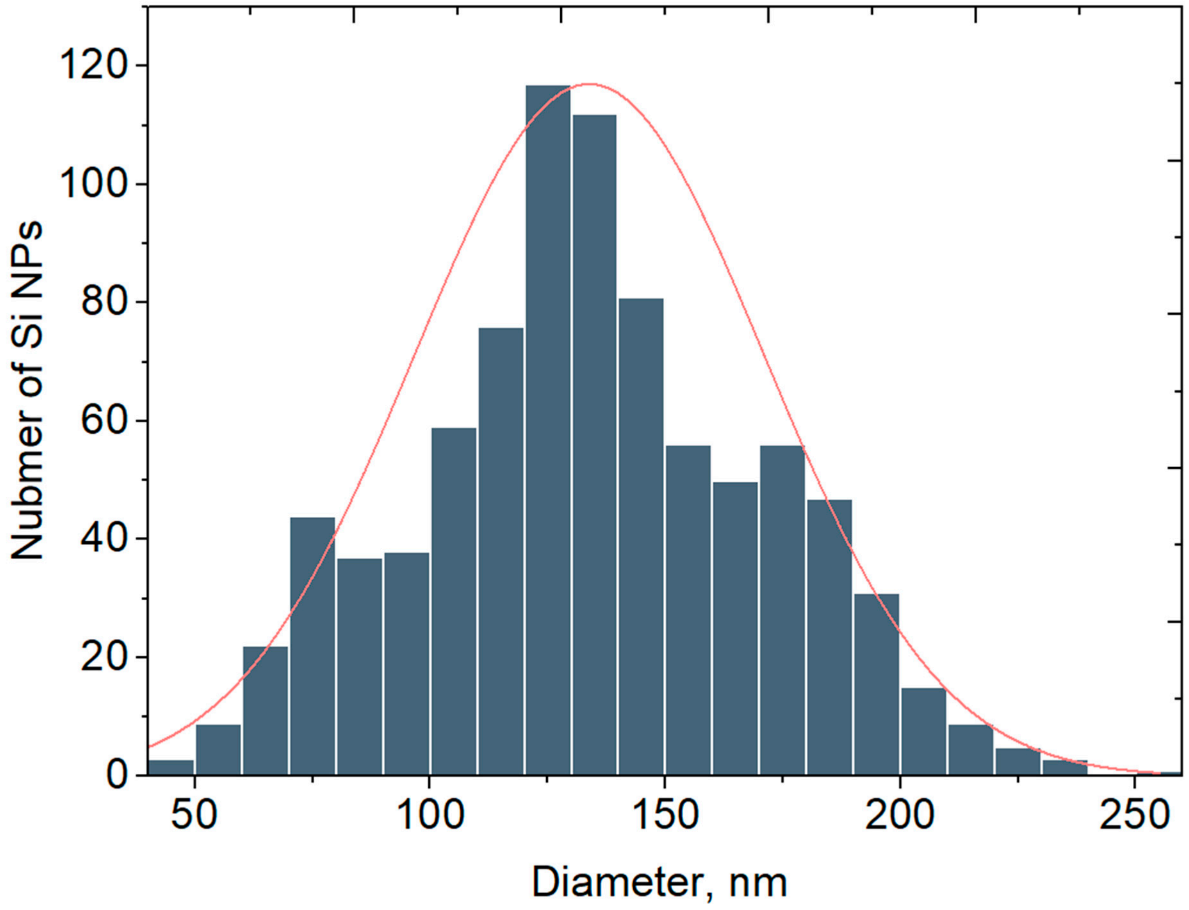

Figure S1. Size distribution. The size distribution of Si nanoparticles measured by SEM.

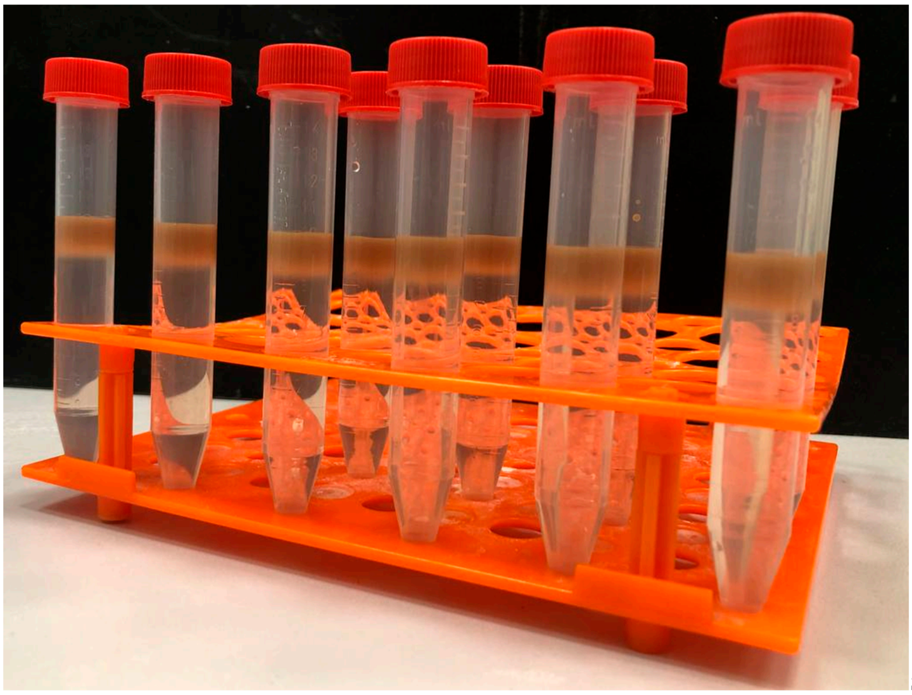

Figure S2. The tubes before centrifugation for mass separation of Si nanoparticles.
